# BLOOD PRESSURE TRAJECTORIES IN YOUTH AND HYPERTENSION RISK IN ADULTHOOD: THE 1970 BRITISH COHORT STUDY

**DOI:** 10.1093/aje/kwz241

**Published:** 2019-11-19

**Authors:** Mark Hamer, Mika Kivimäki, G David Batty

**Affiliations:** 1 Division of Surgery and Interventional Science, Faculty of Medical Sciences, University College London, London, United Kingdom; 2 Department of Epidemiology and Public Health, University College London, London, United Kingdom; 3 School of Biological and Population Health Sciences, Oregon State University, Corvallis, OR

**Table 1:** Crude Risk per 1,000 Person-Years and Odds Ratios for the Association Between Baseline Blood Pressure, Blood Pressure Trajectory During Ages 10–16 Years, and Risk of Hypertension at Age 46 Years^a^, Great Britain, 1980–2018

**BP and Trajectory** ^**b**^	**Total No.**	**No. of Hypertension Cases**	**Risk Per 1,000 Person Years**	**OR** ^**c**^	**95% CI**
BP, age 10 years					
Normal	5,112	672	4.2	1.00	Referent
High	100	30	11.9	2.48	1.60, 3.88
BP trajectory, from ages 10 to 16 years					
Stable, low	346	28	2.4	1.00	Referent
Low + increase	890	111	4.0	1.47	0.95, 2.29
Stable, high	904	132	4.7	1.73	1.12, 2.66
High + increase	346	77	8.0	2.61	1.63, 4.20

Abbreviations: BP, blood pressure; CI, confidence interval; OR, odds ratio.

^a^ Sample sizes vary; *n* = 5,212 had data for ages 10 and 46 years; *n* = 2,486 had data for ages 10, 16, and 46 years.

^b^ Stable, low: systolic BP below the median (100 mm Hg) at baseline and below the median (13 mm Hg) for increase in systolic BP between ages 10 and 16 years; low + increase: baseline SBP of <100 mm Hg and an increase of ≥13 mm Hg; stable, high: baseline SBP of ≥100 mm Hg and change of <13 mm Hg; high + increase: baseline SBP of ≥100 mm Hg and change of ≥13 mm Hg.

^c^ Adjusted for sex, body mass index, sports participation at age 10 years, father’s occupation, and parental smoking.

During the last 10 years, raised blood pressure (BP), including hypertension, has been the leading contributing factor to cardiovascular disease burden globally (1). Current prevention strategies target adults, but intervention earlier in the life course might be needed to significantly reduce cardiovascular disease risk in adults. The degree to which BP tracks from childhood to adulthood varies by sex, age, and length of study follow-up, suggesting that a single BP measurement might not be enough to identify children who could benefit from early interventions (2). Sparse data exist on BP trajectories from this earlier period. In one study, a “high, increasing” trajectory was associated with carotid intima-media thickness and left ventricular mass index in adulthood (3). We hypothesized that repeat BP measurements in childhood would allow identification of BP trajectories that reliably predict adulthood hypertension. Adulthood diabetes, another major risk factor for cardiovascular health, was examined as a secondary outcome.

We used data from the 1970 British Cohort Study of people born in England, Scotland, and Wales in a single week of 1970 (4). Cohort members underwent clinical examinations at ages 10, 16, and 46 years that included measurement of BP using a mercury sphygmomanometer in childhood and an automated Omron HEM-907 monitor (Omron Healthcare, Inc., Bannockburn, Illinois) in adulthood. A total of 5,212 people (2,674 women; 51.3%) had complete data on baseline and adulthood BP, and 2,486 (1,337 women; 53.8%) provided data for all 3 assessments. Close agreement has been shown between the auscultation and oscillometric devices (5).

High BP in childhood was defined using the 95th percentile for systolic and diastolic BP relative to sex and height at age 10 years (6). Hypertension at age 46 years was defined as systolic/diastolic BP of at least 140/90 mm Hg taken from the average of the second and third readings after 5 minutes seated rest, and/or prescribed antihypertensive medication (confirmed from British National Formulary codes on medication dispensers) (7). For analyses using the continuous BP data, a constant of 10 mm Hg was added to systolic and diastolic BP in participants taking antihypertensive medication (8). A diabetes outcome in adulthood was generated from data on physician-diagnosed diabetes and/or glycated hemoglobin of at least 48 mmol/mol (>6.5%).

We generated four BP trajectories using baseline systolic BP at age 10 years and the change in BP between ages 10 and 16. “Stable, low” was defined as systolic BP below the median (100 mm Hg) at baseline and below the median (13 mm Hg) for increase in systolic BP between ages 10 and 16 years; “low + increase” was baseline SBP of <100 mm Hg and an increase of ≥13mm Hg; “stable, high” was baseline SBP of ≥100 mm Hg and change of <13 mm Hg; “high + increase” was baseline SBP of ≥100 mm Hg and change of ≥13 mm Hg.

The association of BP trajectory with hypertension and diabetes outcomes at age 46 years was examined using logistic regression models where the relationship was adjusted for sex, body mass index, sports participation at age 10 years, father’s occupation, and parental smoking. All analyses were conducted using SPSS, version 22 (SPSS Inc., Chicago, Illinois).

At the age-46 follow-up, 702 cases of hypertension and 202 cases of diabetes were recorded. High BP (based on 95th percentiles) was evident in 1.9% of the cohort at age 10 years and, compared with normal BP, was associated with approximately a doubling of hypertension risk at age 46 years (odds ratio = 2.48, 95% confidence interval (CI): 1.60, 3.88) (Table 1), although not with diabetes (odds ratio = 1.24, 95% CI: 0.53, 2.92). Between the assessments at ages 10 and 16 years there was, on average, an increase in both systolic (98.1 vs. 111.1 mm Hg; *P* < 0.001) and diastolic (62.4 vs. 69.3 mm Hg; *P* < 0.001) BP. Participants who had elevated systolic BP at age 10 years that continued to rise above average levels through to age 16 demonstrated a more than 2.6-fold increased odds of hypertension by middle age (Table 1), although no associations were observed for diabetes (odds ratio = 1.64, 95% CI: 0.71, 3.76). Compared with cohort members classified as having a “stable, low” trajectory, systolic BP at age 46 years was elevated by 3.3 mm Hg (95% CI: 1.4, 5.2) in the “low + increase” group, 5.2 mm Hg (95% CI: 3.3, 7.0) in “stable, high” group, and 8.4 (95% CI: 6.2, 10.7) mm Hg in the “high + increase” group after adjusting for covariates. Differences in diastolic BP followed a similar pattern; 2.6 mm Hg (95% CI: 1.1, 4.0) in the “low + increase” group, 3.4 mm Hg (95% CI: 2.0, 4.8) in “stable, high” group, and 5.8 mm Hg (95% CI: 4.1, 7.5) in the “high + increase” group.

In the present study, we have shown that BP in childhood is an important predictor of hypertension risk in middle-aged adulthood. Thus, childhood might be a key period for early intervention. The trajectory in BP from ages 10 to 16 years was further able to discriminate differences in hypertension risk at age 46 (middle age). Although annual BP measurement after 3 years of age is supported by some, such as American Academy of Pediatrics (9), others have not endorsed such recommendations (10). Our findings support recommendations to track BP trajectories in clinical settings.
